# Photocatalytic Activation of Saturated C–H Bond Over the CdS Mixed-Phase Under Visible Light Irradiation

**DOI:** 10.3389/fchem.2018.00466

**Published:** 2018-10-09

**Authors:** Houde She, Liangshan Li, Hua Zhou, Lei Wang, Jingwei Huang, Qizhao Wang

**Affiliations:** ^1^College of Chemistry and Chemical Engineering, Northwest Normal University, Lanzhou, China; ^2^Gansu International Scientific and Technological Cooperation Base of Water-Retention Chemical Functional Materials, Lanzhou, China

**Keywords:** toluene, benzaldehyde, ion modify, mixed-phase, C–H bond, selective oxidation

## Abstract

Selective activation of saturated C–H bond in hydrocarbons to produce high-value-added chemicals is of great significance for chemical synthesis and transformation. Herein, we present a facile procedure to achieve Ni-doped CdS nanoparticles with mixed (cubic and hexagonal) phases, as well as its application to the photocatalytic activation of saturated primary C–H bond of toluene and its derivatives. The photocatalytic oxidation rate of toluene into benzaldehyde of formation reached up to 216.7 μmolh^−1^g^−1^ under visible light irradiation. The excellent photocatalytic performance of Ni(II)-doped CdS [Ni(II)/CdS] can be attributed to its unique structural assembly with cubic and hexagonal phases and also the addition of Ni ions, together taking effect in promoting the separation of photogenerated charge carriers. The possible reaction mechanism for the photocatalytic selective oxidation is illustrated in this work. The band width of the as-prepared mixed phase CdS is reduced, which can effectively expand the response range and improve photocatalytic performance.

## Introduction

The highly selective oxidation of inactive C–H bond in hydrocarbons to form high value-added oxygenated products under mild conditions is one of the major challenges in the industrial field (Pal et al., 2014; Yang et al., 2017). For example, toluene is a simple aromatic compound and can be selectively oxidized into benzaldehyde, benzyl alcohol, benzoic acid and benzyl benzoate, all of which are crucial intermediate for the manufacture of fine chemicals, such as pharmaceuticals, dyes, preservatives and perfume. At present, benzaldehyde is primarily synthesized via traditional route of chlorination hydrolysis of toluene. The discharges of toxic gases and organic waste bring on serious environmental concerns. Therefore, it is essential to develop an environment-friendly technique to address such problem. Photocatalysis is a promising alternative strategy that has been widely applied in the activation of C–H bond under mild conditions (Zhang et al., 2012; Hao et al., 2018) owning to its clean and low energy consumption, etc. For example, Yuan et al. reported that BiOBr/TiO_2_ can be used in oxidation of toluene into benzaldehyde under visible light irradiation (Yuan et al., 2013). He's group (He et al., 2018) presented a toluene selective oxidation that has been carried out by Cd_3_(C_3_N_3_S_3_)_2_/CdS porous composites under visible light irradiation. Cd_3_(C_3_N_3_S_3_)_2_/CdS exhibits excellent performance for the transformation of toluene into benzaldehyde.

It is well-known that the CdS is economical and has been extensively employed in photocatalysis research because of its adequate band gap (2.45 eV), which is unique among photocatalytic materials (Wang et al., 2014; Cheng and Xiang, 2016; Xiang et al., 2016). Although CdS-based photocatalysts are widely studied in the field of photocatalysis (Yan et al., 2009; He et al., 2016b; Li et al., 2018) some inherent defects like high charge carrier recombination rate and photo-corrosion, debase their photocatalytic activity and practical application (He et al., 2016a). To hold back electrons-holes recombination and improve photocatalytic activity, various strategies, such as the loading of co-catalysts, forming heterojunctions have been scrupulously developed (Wang et al., 2013; Wei et al., 2017, 2018). In the past decade, Pt (Luo et al., 2015b), Au (Majeed et al., 2016; Wang et al., 2018b), Pd (Luo et al., 2015a), Ag (Gupta et al., 2017), and Ru (Nosheen et al., 2017) have been used as co-catalysts in various photocatalyst systems to enhance charge separation and surface reactions. Despite the high photocatalytic performance achieved by these noble metal-based co-catalysts contained catalyst, their use in mass production are limited due to the scarcity and high cost of them. Therefore, it is desirable to investigate new catalyst system with non-noble metal as co-catalyst, which can meet the general industrial requirements, such as low cost, high efficiency, and good durability. In this regard, many non-precious metals [such as Fe (Yan et al., 2017; Deka and Kalita, 2018), Cu (Kumar et al., 2018; Wang et al., 2018a), Co (Dong et al., 2017), Ni (Yang et al., 2018), Mo (Zhang et al., 2017)] materials have been studied as a co-catalyst to improve performance of main catalyst. In particular, nickel-based materials [such as metallic nickel, nickel ion (Song et al., 2017), nickel oxides and hydroxides (Zhang et al., 2015)] exhibit excellent activity and attract much attention as promoters for enhancing photocatalytic activity. Murugesan et al. (Murugesan et al., 2017) prepared Ba and Ni doped CdS by a spray pyrolysis method. Doping Ba and Ni can significantly improve optical performance of CdS. Besides, it has also been suggested that Ni doped CdS was prepared via an impregnation method (Yu et al., 2016), which doped samples can efficiently promotes separation of holes and electrons and consequently enhances the photocatalytic hydrogen production.

However, in the above works, only modified single phase CdS can enhance the photocatalytic activity. But, as reported by some researchers, the CdS base can also form a homojunction for enhancing its photocatalytic activity (Dai et al., 2014). The mixed-phase CdS has been reported by Zhao's group (Zhao et al., 2018), which demonstrated that the photoresponse range can be broadened to ameliorate photocatalytic activity.

In this work, we report the synthesis of Ni(II) doped CdS (Ni(II)/CdS) with mixed-phase (cubic and hexagonal) via a simple impregnation method. The Ni(II)/CdS was further used as photocatalyst in the activation of saturated primary C–H bond of toluene and its derivatives at room temperature and under two barometric pressure (0.2 MPa). The yield of benzaldehyde can reach up to 216.70 μmolh^−1^g^−1^, which is 7.7 times higher than that of mixed-phase CdS. Controlled experiments using different radical scavenger were also conducted to elucidate the possible reaction mechanism for the selective oxidation of C–H bond in toluene over the mix phase of Ni(II)/CdS.

## Experimental

### Materials

All reagents were analytical grade and used without further purification. Benzotrifluoride (BTF) (>99%)was purchased from Aladdin Chemical Reagent Co., Ltd. Oleic acid (C_18_H_34_O_2_), Cadmium nitrate tetrahydrate [Cd(NO_3_)_2_·4H_2_O], 8% of ammonium sulfide [(NH_4_)_2_S], sodium borohydride (NaBH_4_), ethyl alcohol (C_2_H_6_O), toluene (>99.5%) and other chemical reagents were purchased from Sinopharm Chemical Reagent Co., Ltd.

### Preparation

#### Synthesis of mixed-phase CdS

Typically, Cd(NO_3_)_2_·4H_2_O (1.0 mmol) and oleic acid (0.0375 ml) were first dissolved in deionized water (50 mL) in a beaker placed on a magnetic stirrer for 15 min at room temperature to form solution A. NaBH_4_ (4.0 mmol) was also dissolved in deionized water (250 mL) in a beaker placed on a magnetic stirrer at room temperature to form solution B. The two solutions were then mixed and quickly put into a microwave oven (M1-L213B, at a fixed frequency of 2,450 MHz, Guangdong midea kitchen appliances manufacturing Co., Ltd., China) followed by 20 s irradiation. The Cd nanoparticles (NPs) were collected and washed by ethyl alcohol three times, and dispersed in 20 mL ethyl alcohol to get sulfurized by adding certain amount of (NH_4_)_2_S (2.0 mmol) and followed by 1 h stirring at room temperature. Then the yellowish products were collected and washed by ethyl alcohol three times. Finally, the products were dried in drying oven at 60°C for 4 h.

#### Preparation of Ni(II)-mixed-phase CdS samples

The Ni(II) doped mixed phase CdS samples (Ni(II)/CdS) were synthesized by a simple impregnation method. Typically, CdS (200.0 mg) was dispersed into10.0 mL of NiCl_2_·6H_2_O solution and stirred at 80°C for 2 h. The products was collected and washed by deionized water several times. The amount of Ni (the weight ratio of Ni(II) to CdS) was about 0.5, 1.0, 3.0, and 5.0 wt%, respectively. Finally, the samples dried in drying oven at 60°C for 4 h.

### Characterization

X-ray diffraction (XRD) patterns were acquired by a Rigaku D/MAX-2200/PCX-ray diffractometer in the angular range of 10–90° (2θ) with Cu Kα radiation (40 kV, 20 mA). UV-vis diffuse reflectance spectra (DRS) were acquired from 230 to 800 nm by a UV-vis spectrophotometer (PuXin TU-1901). The morphologies and microstructures of the samples were obtained using field emission scanning electron microscope (FE-SEM, Ultra Plus, Carl Zeiss) and transmission electron microscope (TEM, F20, FEI). X-ray photoelectron spectroscopy (XPS) was used to study the chemical compositions and the valence states by a photoelectron spectrometer (PHI5702). The photoelectrochemical (PEC) performances of photoanodes were acquired by a three-electrode system (CHI-660D Co., Shanghai, China) under a LED lamp (λ > 420 nm, CEL-LED100) illumination. A Pt wire and Ag/AgCl were used as counter electrode and reference electrode, respectively. The working electrodes were made on the fluride-tin oxide (FTO) conductor glasses. The samples (10 mg) were homogeneously dispersed in anhydrous ethanol and ultrasound for 60 min, which were slowly dripped on FTO glasses. The electrolyte was 0.5 M Na_2_SO_4_ (pH = 7.35) aqueous solution in a quartz ware 0.5 V of the bias voltage was used for photoelectrochemical testing. Illumination through the back-side of FTO was used with an illumination area of about 1.0 cm^−2^. Photoluminescence (PL) spectra of the samples were recorded by a fluorescence spectrophotometer (PE, LS-55) at room temperature, using a 390 nm excitation wavelength.

### Evaluation of photocatalytic activity

The photocatalytic reaction was performed in a 25 mL glass bottle under visible light irradiation in this work. Typically, 0.5 mmol of substrate and 80 mg of catalyst were dispersed in the 5.0 mL of solvent (BTF) in a 25 mL glass bottle. Then the glass bottle was transferred into a closed reactor (Figure S1) and stirred for 30 min in dark to make the catalyst fully contact with the solution and achieve adsorption equilibrium. Meanwhile, the reactor was bubbled into oxygen molecules from a gas cylinder for 30 min at absolute pressure of 0.2 MPa. A 300 W Xe arc lamp (CEL-HXF 300, Beijing Perfectlight Co. Ltd.) with a ultraviolet cutoff filter (λ < 420 nm) was used in the following photocatalytic reaction. After 2 h of irradiation, the mixture was centrifuged to remove catalyst powders at 8,000 rmp for 5 min by a centrifuge (TG16-WS). The liquid supernatant was analyzed by a gas chromatograph (GC-9600). A series of controlled experiments were carried out similar to the photocatalytic oxidation process of toluene, except that the radical scavengers (0.1 mmol) were added to the reaction system. The conversion (Con.) rate of substrates, yield of aldehyde, and selectivity (Sel.) were defined with the following equations:

(1) $$\begin{array}{l} \text{Con}.\left(\text{\%}\right)=\left[\left({\text{C}}_{0}-{\text{C}}_{1}\right)/{\text{C}}_{0}\right]\times 100\% \end{array}$$

(2) $$\begin{array}{l} \text{Yield}\left(\text{\%}\right)=\left({\text{C}}_{\text{aldehyde}}/{\text{C}}_{0}\right)\times 100\% \end{array}$$

(3) $$\begin{array}{l} \text{Sel}.\left(\text{\%}\right)=\left[{\text{C}}_{\text{aldehyde}}/\left({\text{C}}_{0}-{\text{C}}_{1}\right)\right]\times 100\% \end{array}$$

Where C_0_ is the initial concentration of substrates; C_1_ and C_aldehyde_ are the concentration of the remaining substrates and the corresponding aldehyde at a certain time after the photocatalytic reaction, respectively.

## Results and discussion

### DRS, XRD and photoluminescence (PL) spectra analysis

**Figure 1:**
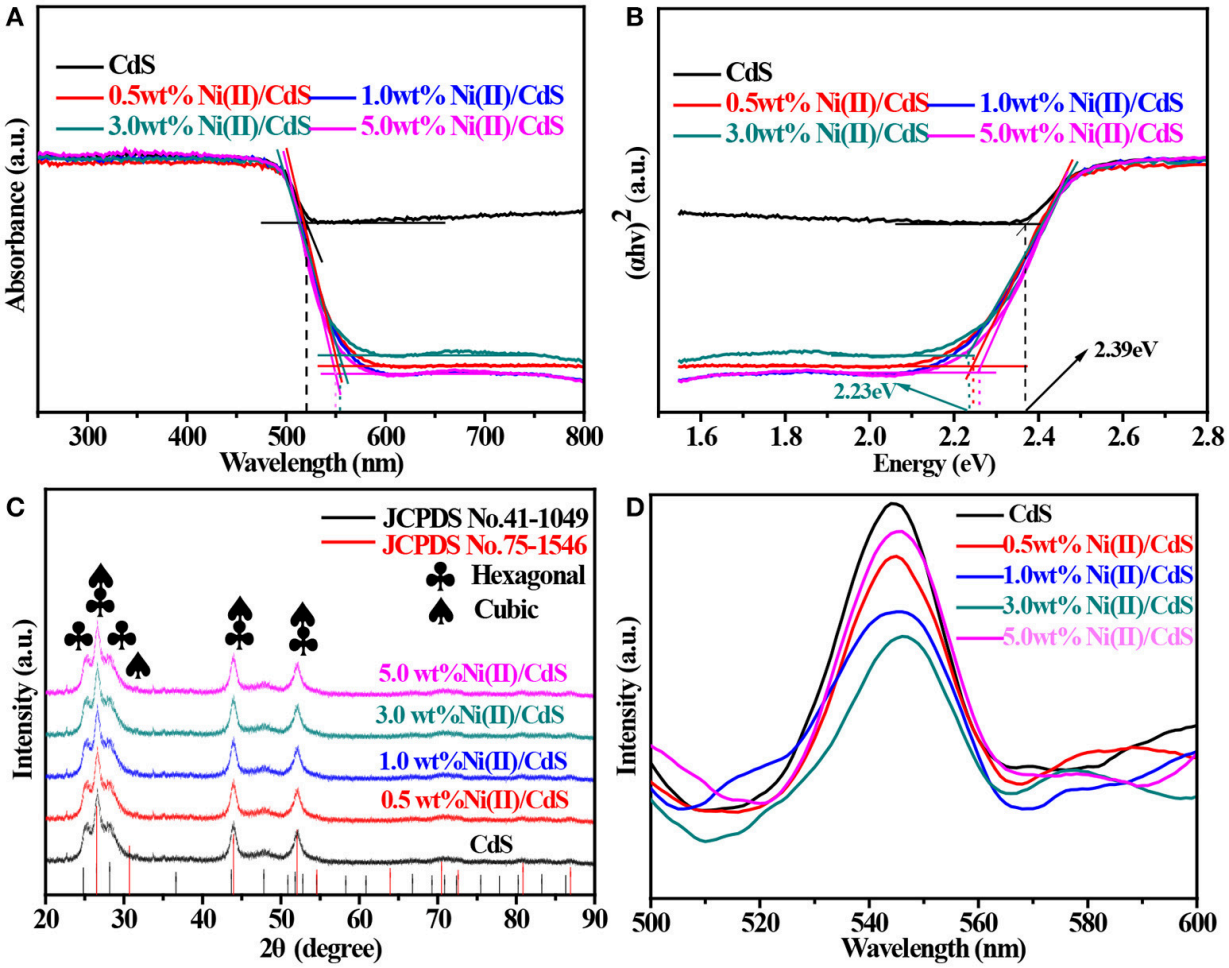
**(A)** UV–vis diffuse reflectance spectra, **(B)** Tauc plots, **(C)** XRD patterns and **(D)** PL of the samples: mixed-phase CdS, 0.5 wt%Ni(II)/CdS, 1.0 wt%Ni(II)/CdS, 3.0 wt%Ni(II)/CdS, and 5.0 wt% Ni(II)/CdS.

Figure 1A shows the UV–vis diffuse reflectance spectra of mixed-phase CdS, 0.5 wt%Ni(II)/CdS, 1.0 wt%Ni(II)/CdS, 3.0 wt%Ni(II)/CdS, 5.0 wt%Ni(II)/CdS samples. All samples of the spectra display absorption in the visible-light region. The mixed-phase CdS NPs exhibit a absorption edges at 518 nm, while the Ni(II)/CdS samples show red shift of absorption edges compared with prestine CdS sample. In particular, the absorption edges of 3.0 wt%Ni(II)/CdS NPs was observed at 555 nm. Hence, it is clear that the modification of Ni(II) co-catalyst can significantly affect the optical absorption feature of CdS NPs. The optical band gap value has been calculated via the equation: (αhv)^2^ = A(hv — Eg) (Su et al., 2013; Kuang et al., 2015), where α, h, A and v correspond to absorption coefficient, Planck constant, proportionality and light frequency, respectively. As shown in Figure 1B, the band gaps estimated are 2.39, 2.26, 2.24, 2.23 and 2.26 eV for mixed-phase CdS, 0.5 wt%Ni(II)/CdS, 1.0 wt% Ni(II)/CdS, 3.0 wt%Ni(II)/CdS and 5.0 wt%Ni(II)/CdS, respectively. The above results indicate that the Ni(II)/CdS have changed the band gap of the mixed-phase CdS. Compared with the single-phase CdS of absorption edge, the absorption edge of the mixed phase CdS is red shift. Prior to this, the cubic CdS was synthesized by a solvothermal method. In addition, the UV–vis diffuse reflectance spectra and Tauc plot are shown in Figure S2.

The X-ray diffraction (XRD) pattern of as-prepared CdS sample is shown in Figure 1C. It is evident that the sample contain hexagonal phase CdS because the peaks at 24.8, 26.5, and 28.2° correspond to the (100), (002) and (101) crystal planes (hexagonal CdS, JCPDS No. 41–1049), respectively. Meanwhile, peaks at 26.5, 44.0 and 52.1° can be indexed to (111), (220), and (311) planes of cubic phase CdS (JCPDS No. 75-1546) or (002), (110), and (112) planes of hexagonal phase CdS. So it is clear that most of the strong diffraction peaks of JCPDS No. 75–1546 (cubic phase) overlap with the peaks of JCPDS No. 41–1049 (hexagonal phase) except for the peak at 30.7°, which corresponds to (200) plane of cubic phase CdS. But it is not safe to judge the existence of cubic phase CdS from the XRD patterns simply according to this distinctive peak because the intensity of (200) peak is only one fifth of that of (111) plane due to its weak and even indiscernible signal in the whole pattern. However, the nature of mixed phases of our samples can be alternatively judged from the intensity distribution of diffraction peaks, as suggested by the peak of 26.5° corresponds to the (111) crystal plane and overlaps with the (002) crystal plane of the hexagonal phase. Noting that peak of (002) plane is not the strongest line in JCPDS No. 41-1049, it is unreasonable to conclude that the strongest peak is given at 26.5° if our samples are hexagonal phase CdS, the only possible explanation is that our sample should be mixed-phase CdS so that the diffraction wave of (111) plane (cubic CdS) will stack up with that of (002) plane (hexagonal CdS) and presents the strongest peak at 26.5°. Another possibility for the presence of the strongest peak at 26.5° can be explained by anisotropic growth of hexagonal phase CdS. The dominant growth along (002) plane will give similar result even if our sample is not mixed-phase CdS. To rule out this possibility, SEM and TEM were performed on all the samples and the results (shown in Figure **3**) do not support this supposition. So it is now safe to demonstrate that the CdS sample should be composed of cubic CdS and hexagonal CdS. According to the patterns shown in Figure 1C, the same diffraction peak intensity and width as mixed-phase CdS suggests that the appearance and crystallite size of CdS NPs are not affected by doping different amount of Ni(II). Photoluminescence (PL) spectroscopy is an effective method to study the electronic structure and optical properties of semiconductor materials. Figure 1D shows the PL spectra of all samples at the excitation wavelength of 390 nm in room temperature. It can be seen from Figure 1D that the catalyst samples have a strong signal peak at a wavelength of about 540 nm. In general, a high intensity of the photoluminescent signal indicates a high recombination probability of photo-generated electrons (e^−^) and holes (h^+^) as well as a low photocatalytic activity (Martha et al., 2013; Wu et al., 2014; Weng et al., 2016; Cui et al., 2017a; Nie et al., 2018; She et al., 2018). In this regard, it is seen from Figure 1D that the activity of the catalyst 3.0 wt%Ni(II)/CdS is significantly higher than that of the other samples, which is in a good agreement with the result of catalyst activity tests.

### Morphology and microstructures analysis

**Figure 2:**
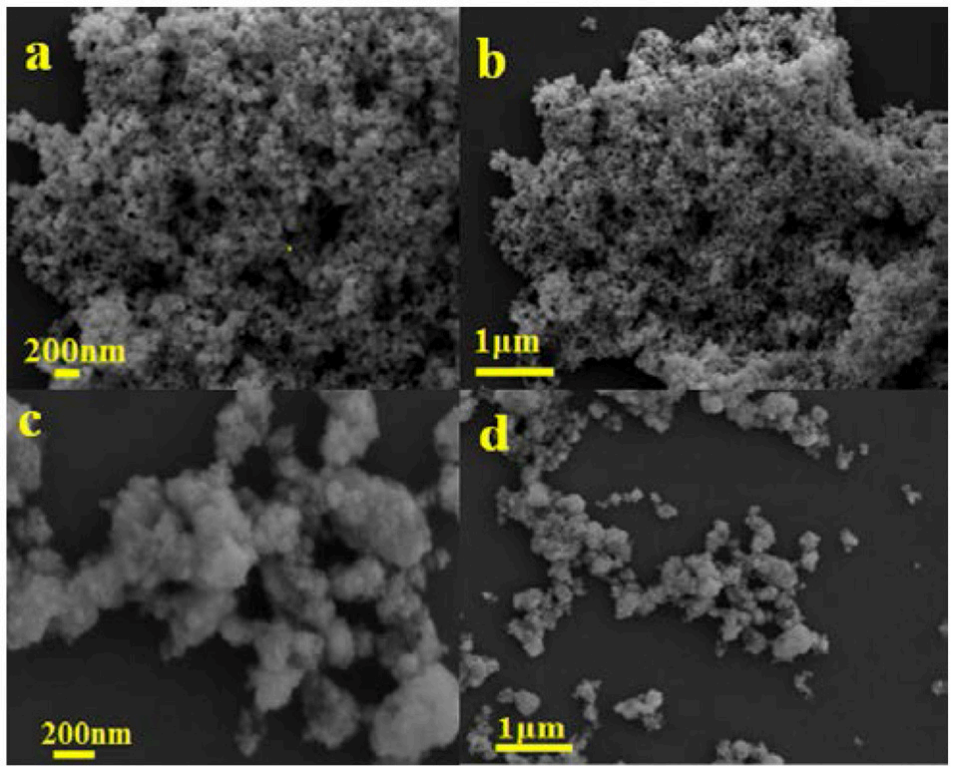
SEM images of the mixed-phase CdS **(a,b)**, and 3.0 wt% Ni(II)/CdS **(c,d)**.

**Figure 3:**
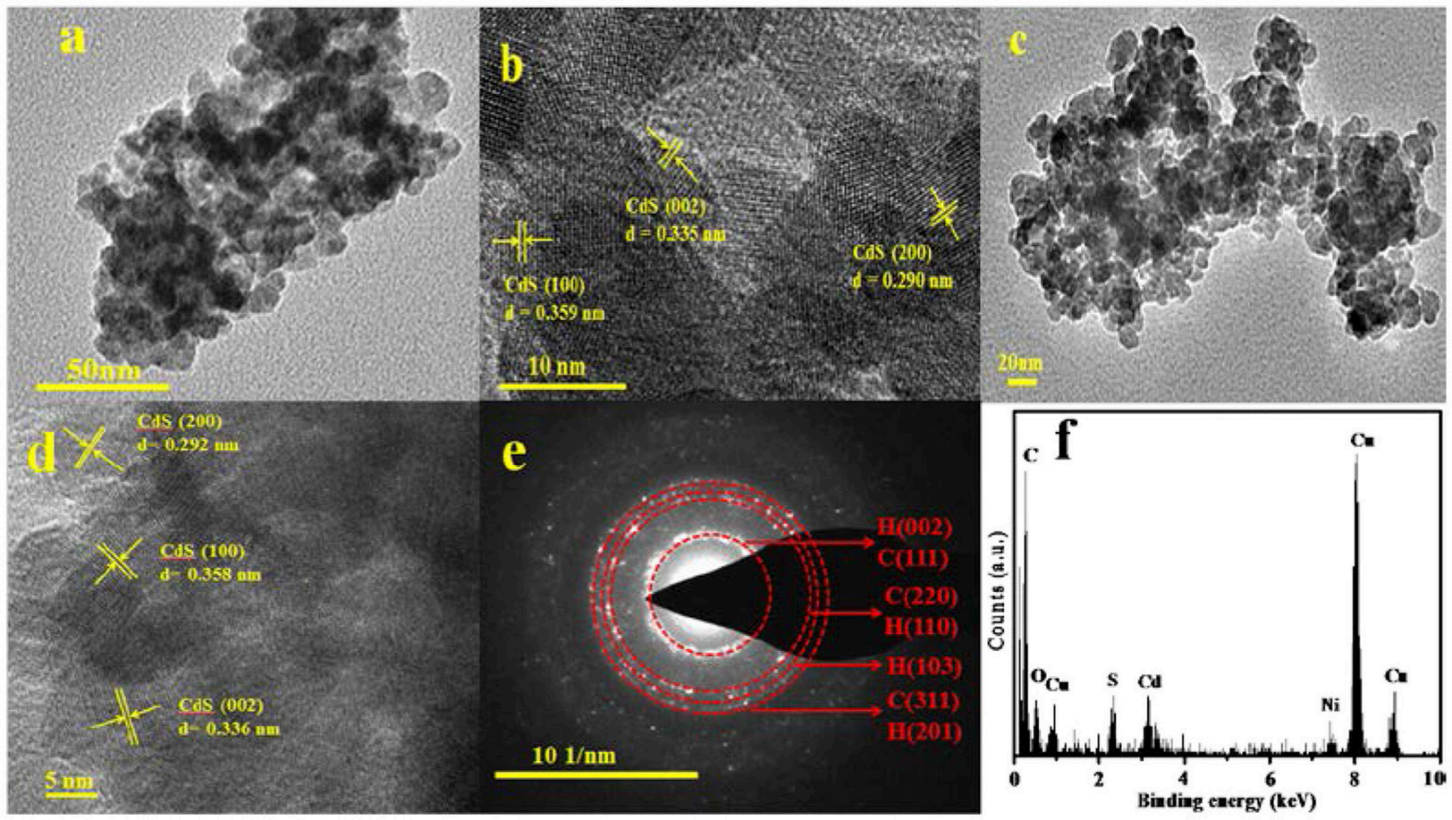
**(a)** TEM image of the mixed-phase CdS, **(b)** HRTEM image of the mixed-phase CdS, **(c)** TEM image of 3.0 wt% Ni(II)/CdS, **(d)** HRTEM image of 3.0 wt% Ni(II)/CdS, **(e)** SAED pattern of 3.0 wt% Ni(II)/CdS, and **(f)** EDX of 3.0 wt% Ni(II)/CdS.

Figure 2 shows the SEM images of the mixed-phase CdS and 3.0 wt%Ni(II)/CdS. It is seen that the mixed-phase CdS sample consists of irregular particles with a size range of 20–100 nm in Figures 2a,b. As exhibited in the Figures 2c,d, 3.0 wt%Ni(II)/CdS sample is also constituted of irregular nanoparticle, and its microscopic structure is similar to that of mixed-phase CdS. But its surface is not so smooth as that of mixed-phase CdS due to the modification of Ni(II). The EDX analysis was also performed to confirm the existence of Ni element and the result is shown in Figure 3f. The SEM images of the 0.5 wt%Ni(II)/CdS, 1.0 wt% Ni(II)/CdS and 5.0 wt% Ni(II)/CdS are displayed in Figure S3, respectively. It can be clearly seen that all the samples are constituted of irregular nanoparticle with the same microscopic structure.

To study the microscopic structure and morphology of as-prepared catalysts, TEM, HRTEM, SAED, and EDX analysis were performed and the results are shown in Figure 3. As shown in Figure 3b, the lattice spacing of 0.359 and 0.335 nm can be ascribed to the (100) and (002) crystal face of hexagonal phase and the spacing of 0.290 nm can be ascribed to the (200) crystal face of cubic CdS. This image analysis further confirms that the as-prepared catalysts are composed of cubic and hexagonal phase CdS NPs and is consistent with the XRD results as discussed above. Compared with prestine CdS (Figure 3a), it is observed that the microscopic structure and morphology of 3.0 wt%Ni(II)/CdS (Figure 3c) is not significantly changed. The lattice spacing of Ni is not observed in Figure 3d, which could be attributed to the low dosage of Ni(II) ions. As shown in Figure 3e, the SAED pattern shows that the sample possesses a polycrystalline nature. In addition, the energy dispersive X-ray (EDX) analysis also suggests the coexistence of Cd, S and Ni elements in Figure 3f.

### XPS analysis

**Figure 4:**
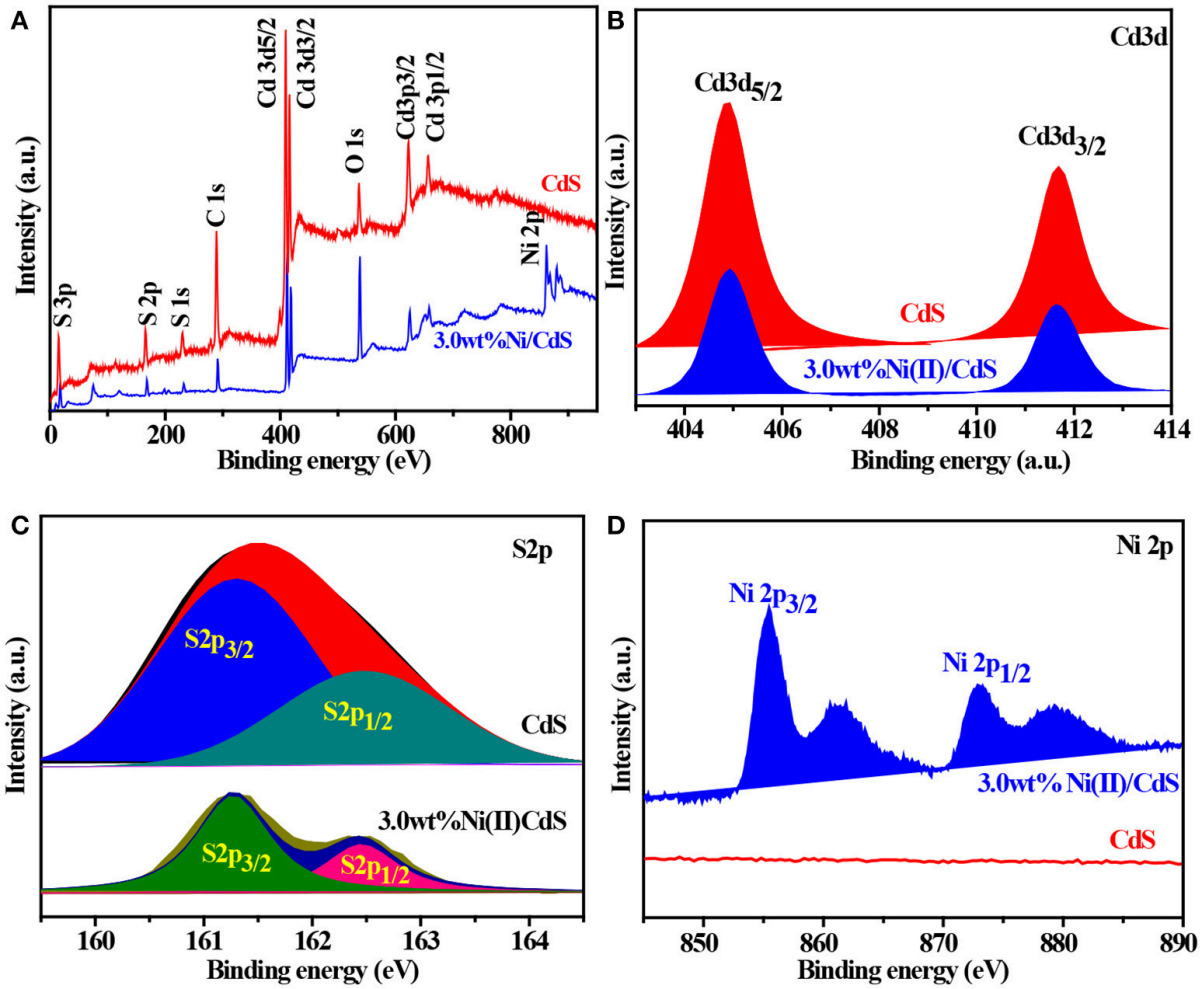
**(A)** XPS survey spectra and the high-resolution XPS spectra of **(B)** Cd 3d, **(C)** S 2p, and **(D)** Ni 2p for the all samples.

The surface nature of the as-prepared CdS and 3.0 wt%Ni(II)/CdS samples were characterized by XPS measurements. As shown in Figure 4A, Cd and S elements are derived from the CdS phase, while the O element might come from H_2_O (Chen et al., 2015). We can see that the peaks at the binding energy of 404.9 and 411.7 eV correspond to Cd 3d_5/2_ and Cd 3d_3/2_ in Figure 4B (Jin et al., 2015). Figure 4C indicates the S 2p peak bifurcates as two peaks of S 2p_3/2_ and S 2p_1/2_ (Xue et al., 2018), corresponding to the binding energy of 161.4 and 162.7 eV (Li et al., 2015b), respectively. As shown in the Figure 4D (3.0 wt%Ni(II)/CdS), the peaks at about 855.5 and 873.1 eV are attributed to Ni 2p_3/2_ and Ni 2p_1/2_ of Ni(II) (Song et al., 2017), with a spin-energy separation of 17.6 eV. The peaks at 861.3 and 879.3 eV are ascribed to the satellite peaks of Ni 2p_3/2_ and Ni 2p_1/2_, respectively (Oros-Ruiz et al., 2014, 2016). The above results also suggest that Ni(II) was successfully decorated on the surface of the CdS nanoparticles.

### Electrochemical characterization

**Figure 5:**
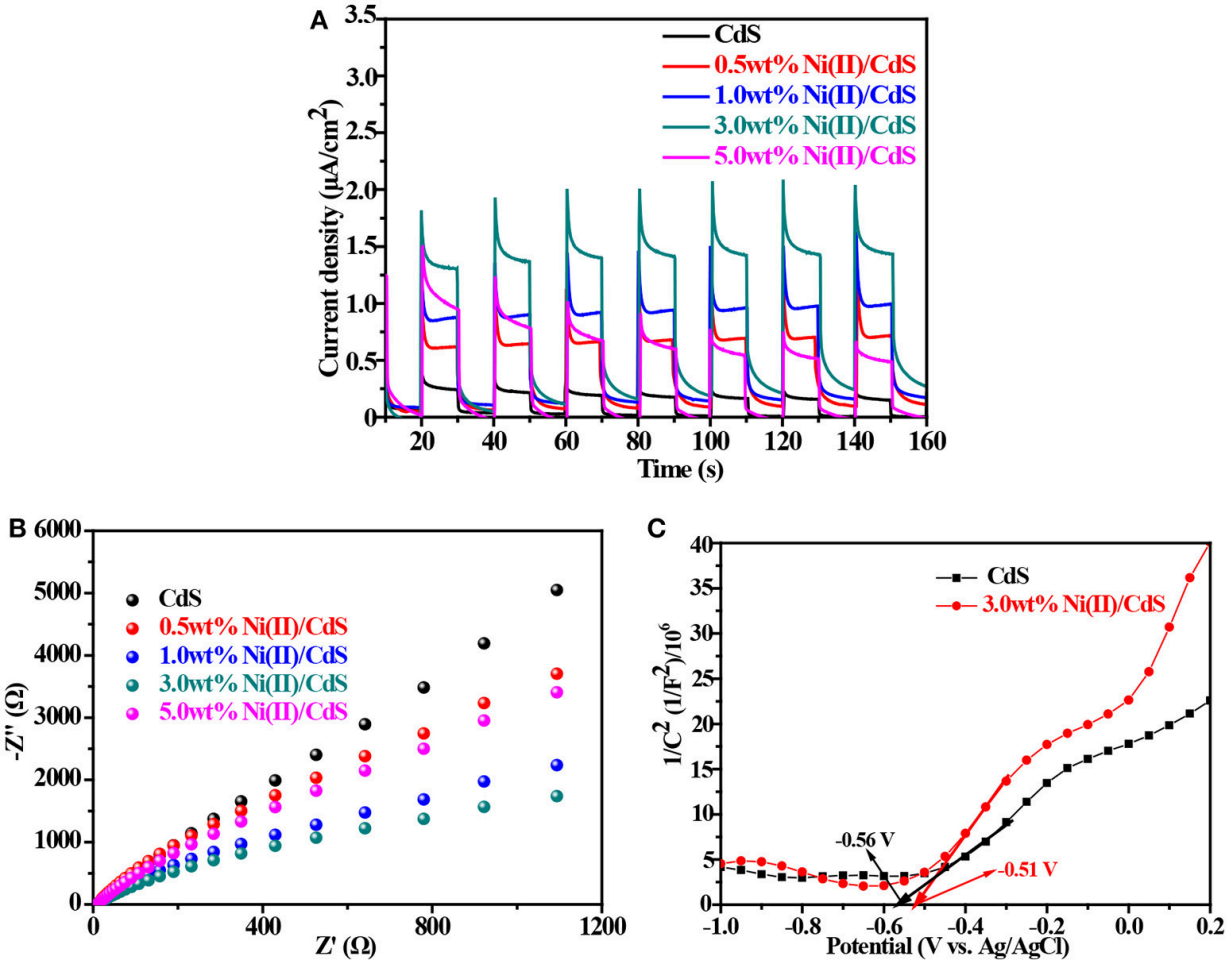
**(A)** Transient photocurrent response. **(B)** Electrochemical impedance profiles. **(C)** Mott-Schottky curves of the samples.

To study the efficiency of the photogenerated electron-hole, the transient photocurrent tests were carried out on all as-prepared samples (mixed-phase CdS, 0.5 wt%Ni(II)/CdS, 1.0 wt%Ni(II)/CdS, 3.0 wt%Ni(II)/CdS and 5.0 wt%Ni(II)/CdS). As shown in Figure 5A, it is clear that all Ni(II)-doped samples have a higher photocurrent than that of prestine CdS, indicating that the separation efficiency of photoinduced charge carriers is improved by doping Ni(II) ions into CdS lattice. The results are consistent with the PL test results (Figure 1D; Zou et al., 2016). Figure 5B shows the electrochemical impedance spectroscopy (EIS) curves of the mixed-phase CdS and Ni(II)/CdS. Comparing the Nyquist plots circle radius of the mixed-phase CdS with that of 3.0 wt% Ni(II)/CdS, it can be observed that the radius of arc curve of 3.0 wt%Ni(II)/CdS is the shortest among all tested samples, suggesting that Ni(II) ions in CdS lattice can facilitate electron-hole separation and interfacial migration (Cui et al., 2017b). It can be also inferred that the Ni(II) ions act as a carrier for transferring electrons. Figures 5C, Figure S4 show the Mott-Schottky curves of the as-prepared samples. The slopes of the two Mott-Schottky curves are all positive in the range of −0.5 and 1.2 V, indicating that the samples are n-type semiconductor, which is consistent with the reported results. The flat band potentials of CdS and 3.0 wt%Ni (II)/CdS are −0.56 and −0.51 V, respectively. The flat band potential is approximately equal to the conduction band (CB) edge for n-type semiconductors. Therefore, the CB positions of CdS and 3.0 wt%Ni (II)/CdS are approximately −0.56 and −0.51 V, respectively. The valence band (VB) edge of samples can be determined by the following equation: Eg = E_VB_ – E_CB_ (Jin et al., 2015; Wang et al., 2018c), where E_CB_ is the CB edge potential, E_VB_ is the VB edge potential, and Eg is the band gap of the semiconductors. The calculated VB positions of CdS and 3.0 wt%Ni (II)/CdS are approximately 1.83 and 1.72 V, respectively.

### Photocatalytic performance experiment

To study the effect of amount of doping Ni(II) on the conversion level, we have tested the photocatalytic performance of the mixed-phase CdS, 0.5 wt%Ni(II)/CdS, 1.0 wt%Ni(II)/CdS, 3.0 wt%Ni(II)/CdS, and 5.0 wt%Ni(II)/CdS for selective oxidation of toluene under visible light irradiation for 2 h. It can be seen from Table 1 that the formation rate of benzaldehyde can be improved when the amount of Ni(II) increases from 0.5 to 3.0 wt%. The formation rate of benzaldehyde can reach to 216.70 μmolh^−1^g^−1^ using 3.0 wt%Ni(II)/CdS as catalyst after 2 h irradiation. The result suggest that 3.0 wt%Ni(II)/CdS is highly active visible-light-driven photocatalyst for the selective oxidation of primary C–H bond in toluene. As we known, metal ions may become the capture center of photogenerated electrons, and effectively suppress the recombination of photogenerated electrons and holes, thereby improving the photocatalytic activity. As the doping amount, the trapping sites of the carriers increase, which increases the carrier lifetime and improves the separation of photogenerated electron-holes. When the doping amount is increases, the carrier capture sites are increased and the carrier lifetime is extended, resulting in a great improvement in photoelectron-hole separation. Therefore, this process enhances the photocatalytic activity. However, when the doping amount is increased to a certain extent, the distance between the trapping sites of the trapping carriers becomes small, the doping ions evolve into a recombination center of electrons and holes, and the activity of the catalyst is lowered. However, when the doping amount is increased to a certain amount, the distance between the trapping sites of the trapping carriers becomes small, resulting in the doping ions evolve into a recombination center of electrons and holes. So, the photocatalyst exhibits lower photocatalytic activity. When the doping amount of 3 wt% is an optimum value, the photocatalyst exhibits the best photocatalytic activity.

**Table 1:** Oxidation of toluene over the as-prepared samples under visible light irradiation for 2 h.

**Entry**	**Catalyst**	**Con. (%)**	**Formation rate (μmolh^−1^g^−1^)**	**Sel. (%)**
1	CdS	3.0	27.93	100
2	0.5 wt%Ni(II)/CdS	7.7	171.08	100
3	1.0 wt%Ni(II)/CdS	8.3	189.08	100
4	3.0 wt%Ni(II)/CdS	8.8	216.70	100
5	5.0 wt%Ni(II)/CdS	7.8	179.20	100

To demonstrate the general applicability of the as-prepared Ni(II)/CdS photocatalysts for selective oxidation of such primary C–H bond in alkyl aromatics, the visible light photoactivity of 3.0 wt%Ni(II)/CdS toward the selective oxidation of other substituted toluenes were further tested, and the results are listed in Table 2. It is clear to see that the 3.0 wt%Ni(II)/CdS is also active for the oxidation of primary C–H bond of substituted toluenes to the corresponding aldehydes. The above photoactivity results suggest that the as-prepared 3.0 wt%Ni(II)/CdS, that is achieved by such a very simple method at room temperature, can be used as an efficient and visible-light-driven photocatalyst toward the selective activation of primary C–H bond in a variety of substituted toluenes.

**Table 2:** Oxidation of toluene and substituted toluenes over the 3.0 wt%Ni(II)/CdS under visible light irradiation for 2 h.

**Entry**	**Substrate**	**Product**	**Con. (%)**	**Formation rate (μmolh^−1^g^−1^)**	**Sel. (%)**
**1**	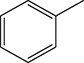	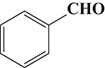	8.8	216.70	100
**2**	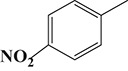	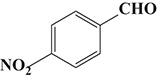	3.5	86.19	100
**3**	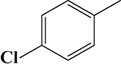	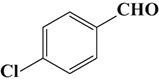	6.4	157.60	100
**4**	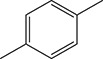	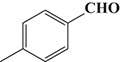	7.1	174.84	100
**5**	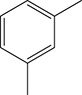	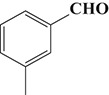	6.1	150.21	100
**6**	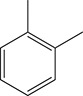	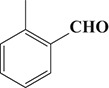	3.4	83.73	100
**7**	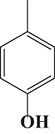	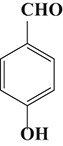	3.6	88.65	100

### Cycle and possible reaction mechanism experiment

**Figure 6:**
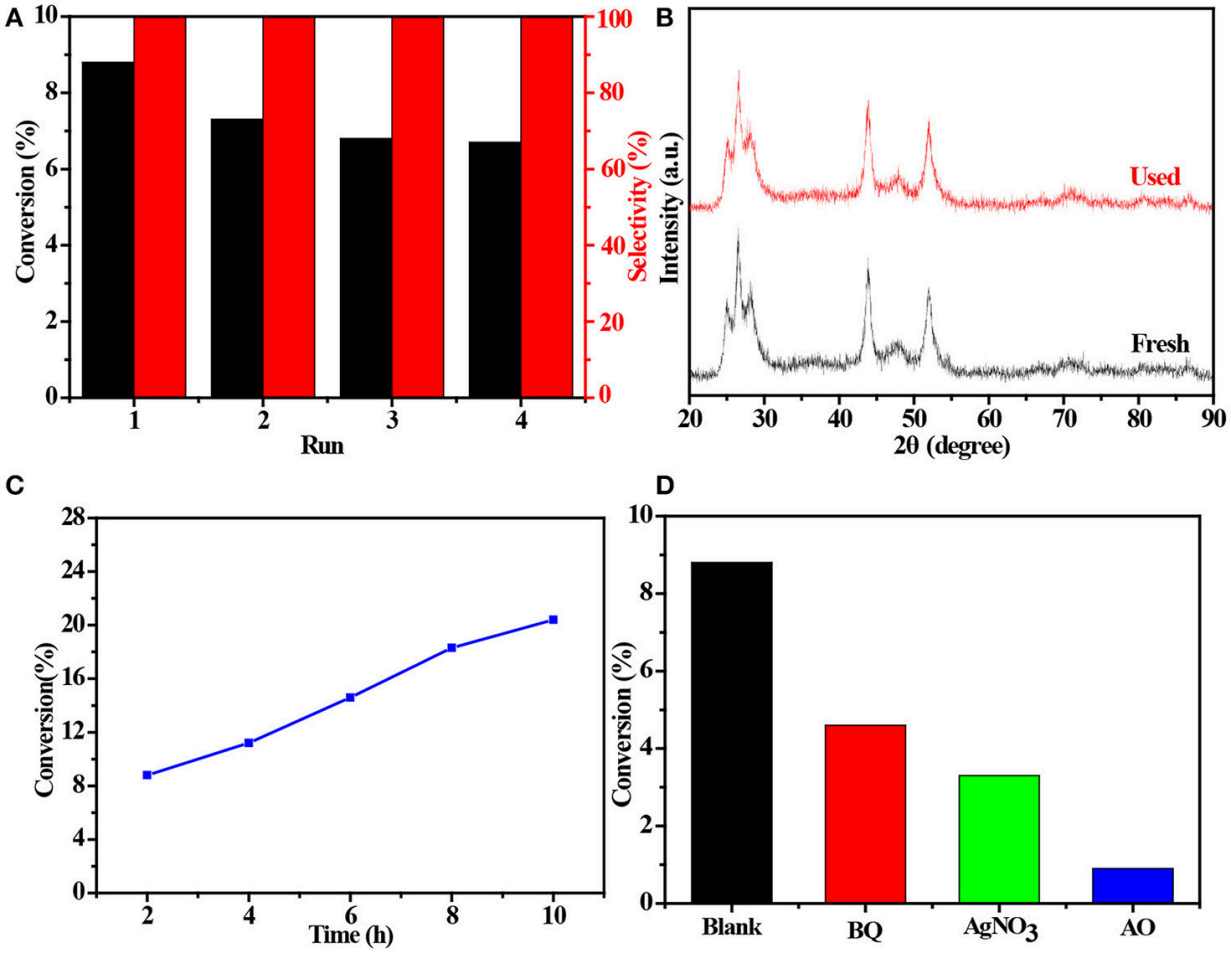
**(A)** Cyclic experiments of 3.0 wt%Ni(II)/CdS to the selective oxidation of toluene to benzaldehyde under visible light irradiation for 2 h, **(B)** XRD patterns of fresh and used 3.0 wt%Ni(II)/CdS after photocatalytic reaction, **(C)** conversion of toluene under different irradiation time over 3.0 wt%Ni(II)/CdS and **(D)** control experiments using different radical scavengers for the photocatalytic selective oxidation of toluene over 3.0 wt%Ni(II)/CdS under visible light irradiation for 2 h.

The 4 times cycling test was performed over the 3.0 wt%Ni(II)/CdS to verify its stability under the same conditions. After irradiation, the reaction mixture was centrifuged to separate the catalyst, which was then washed three times with ethanol and deionized water and dried before next cycle test. In Figure 6A, it can be seen that the conversion of tolueneand the selectivity of benzaldehyde are almost unchanged. To further confirm the stability of our sample as catalyst, the XRD test were performed to measure the fresh 3.0 wt%Ni(II)/CdS as well as the used 3.0 wt%Ni(II)/CdS. As shown in Figure 6B, the crystal structure of 3.0 wt%Ni(II)/CdS sample does not change significantly after the photocatalytic reaction. Furthermore, considering the XRD test on stability of our sample together with the SEM images (shown in Figure S5) of used the 3.0 wt%Ni(II)/CdS after photocatalytic reaction, it can be inferred that the morphology and structure of the sample as photocatalyst remain unchanged during reaction, indicating that the 3.0 wt% Ni(II)/CdS composite is a stable photocatalyst for the selective oxidation of substituted toluenes to the corresponding aldehydes under the experimental conditions. Furthermore, the valence of Ni and its effect on the photocatalytic process through XPS detection of the 3.0 wt%Ni(II)/CdS after photocatalytic reaction had been analyzed. According to XPS (Figure S6), Cd and S present undetectable change between fresh and used 3.0 wt%Ni(II)/CdS. As shown in Figure S6D, two weak peaks at 850.6 and 868.9 eV can be respectively attributed to the 2p_3/2_ and 2p_1/2_ peaks of Ni metal (Li et al., 2015a), indicating the formation of the nickel metallic state of the catalyst during the photocatalytic reaction. It is considered that photogenerated electrons in the CdS CB tended to transfer to Ni(II) clusters and then effectively reduce a portion of Ni(II) to Ni^0^ atoms (Weng et al., 2017). However, the combination of electrons and oxygen molecules quickly forms a process of the long-term transport of electrons. Therefore, more holes are combined with toluene to form an oxidation reaction process. This process facilitates efficient separation of carriers to ameliorate photocatalytic activity. As shown in Figure 6C, toluene oxidation over 3.0 wt%Ni(II)/CdS under visible light irradiation for 10 h can give a toluene conversion rate of 20.4%. It presents a great potential for the industrial synthesis for the fine chemicals. To study the role of photogenerated radical species involved in photocatalytic oxidation of toluene on 3.0 wt%Ni(II)/CdS sample under visible light irradiation and the reaction mechanism involved, a series of control experiments were carried out and the result is shown in Figure 6D. In the control experiments, all conditions were kept unchanged except that different scavengers (AO, BQ and AgNO_3_) were added to capture h^+^, •${\text{O}}_{2}^{-}$ and e^−^, respectively. As shown in Figure 6D, ammonium oxalate (AO) scavenger was added and the conversion of toluene was almost terminated under visible light irradiation. Adding benzoquinone (BQ) or AgNO_3_ to the reaction system also significantly suppressed the conversion of toluene. Although electrons cannot directly participate in the oxidation of toluene, it can affect the activation of oxygen molecules. A series of control experiments result suggest that the h^+^ play the important role in photocatalytic oxidation of toluene over 3.0 wt%Ni(II)/CdS. The O_2_ is activated by e^−^ to form the superoxide radicals, which acts as the main oxidant for the photocatalytic toluene process. The above results suggest that the major active species for the photocatalytic selective oxidation of toluene is h^+^ rather than e^−^.

**Figure 7:**
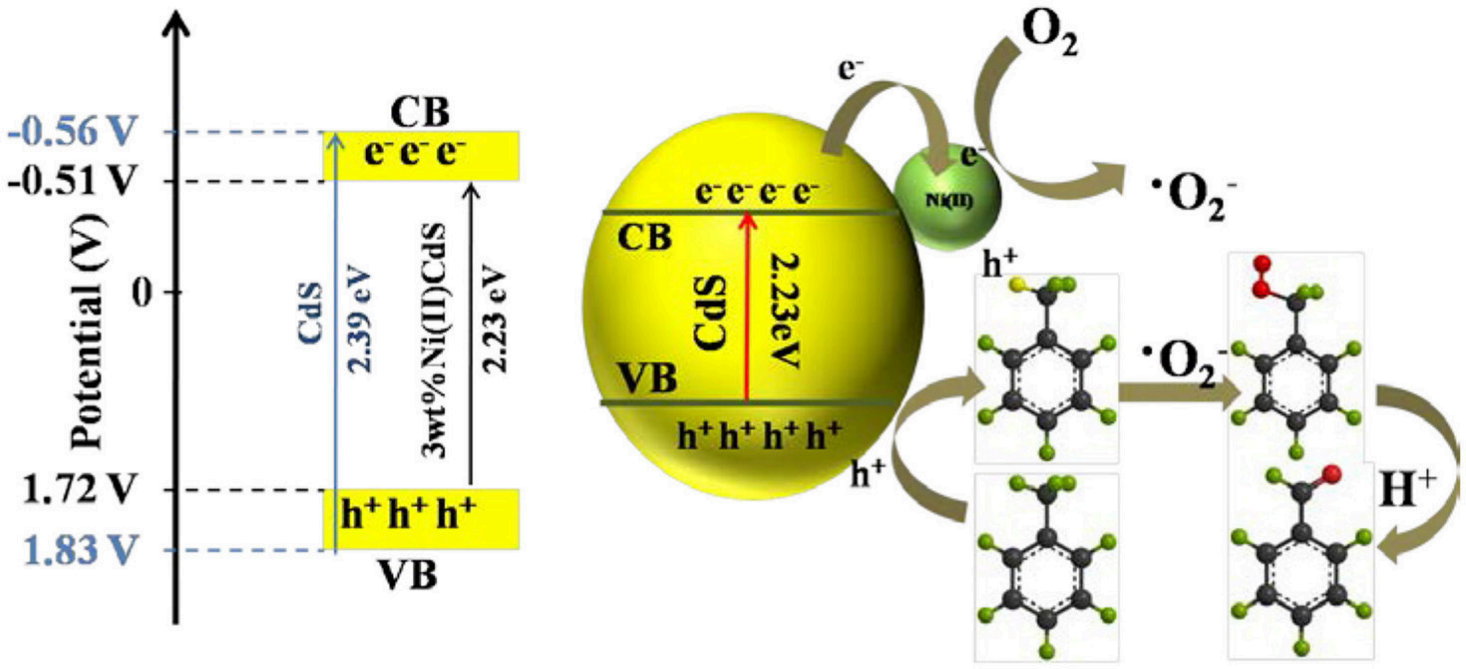
Schematic diagram of the proposed mechanism for oxidation of toluene into benzaldehyde over 3.0 wt%Ni(II)/CdS under irradiation for 2 h.

Thus, the possible mechanism is proposed, as given in Figure 7. Compared with the common CdS of band width (2.41 eV), the band width (2.39 eV) of the mixed phase CdS is reduced. Besides, doping Ni(II) to the photocatalyst is capable of greatly enhancing its electron transport and charge separation. The photogenerated electrons of CdS can be rapidly transferred to the Ni(II) promoter because of the positive potential of Ni^2+^/Ni^0^ (−0.23 V vs. SHE, pH = 0) than the CB potential of the mixed-phase CdS (about −0.56 V, Figure 5C; Ran et al., 2011; Meng et al., 2018), thereby forming a long-term electron transfer process. The toluene is adsorbed on the surface of the 3.0 wt%Ni(II)/CdS and oxidized by the holes to the corresponding cationic radicals. Meanwhile, the electrons react with adsorbed O_2_ to give activated oxygen species. The activated oxygen species then selectively oxidize the cationic radicals, finally leading to the formation of benzaldehyde.

According to the experimental and simulated results, the photocatalytic mechanism of oxidation of toluene coupling with was proposed as following scheme:

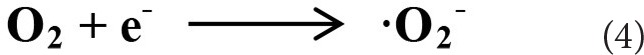

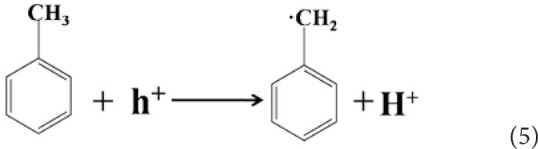

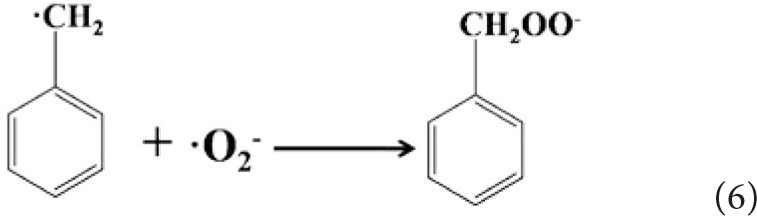

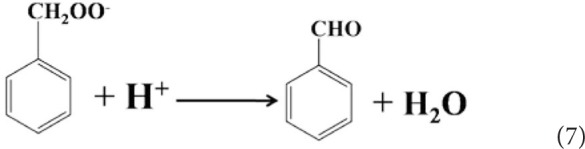









## Conclusion

In summary, we have synthesized Ni(II)-doped cubic and hexagonal phases CdS semiconductor with nanosphere structure morphology by a simple method, which is able to be used as a well-photocatalyst to the activation of saturated primary C–H bond in toluene and substituted toluenes under mild conditions. The mixed phase CdS forms a homojunction, resulting in a reduction in its band width. It can effectively expand the response range and improve photocatalytic performance. Besides, doping Ni(II) to the photocatalyst is capable of greatly enhancing its electron transport and charge separation. The superior photocatalytic performance of Ni(II)-doped CdS is attributed to its unique structure assembly of specific morphology, which can be efficient transport and separation of photogenerated charge carriers under visible light irradiation. Mechanism research shows that Ni as a co-catalyst can improve the catalytic activity of the CdS. The as-prepared 3.0 wt%Ni(II)/CdS has highly active for the selective oxidation of inert primary C–H bond, which has great potential in photocatalytic selective activation of C–H bond to fine chemicals.

## Author contributions

HS and LL designed and conducted the experiments. LL, HZ, and JH analyzed the data. HS, LL, LW, and QW wrote the paper.

### Conflict of interest statement

The authors declare that the research was conducted in the absence of any commercial or financial relationships that could be construed as a potential conflict of interest.
