# Supplementary material for: Photocatalytic Activation of Saturated C–H Bond Over the CdS Mixed-Phase Under Visible Light Irradiation
Source: Front Chem. 2018 Oct 9;6:466. doi: 10.3389/fchem.2018.00466 (PMC6191726; doi:10.3389/fchem.2018.00466)
Supplement: Supplementary file 1 [file Table_1.doc]

Supporting Information

Photocatalytic activation of saturated C-H bond over the CdS mixed-phase under visible light irradiation

Houde She^a^, Liangshan Li^a^, Hua Zhou^a^, Lei Wang^a^, Jingwei Huang^a^, Qizhao Wang^a,b^*

*^a^College of Chemistry and Chemical Engineering,* *Northwest Normal University, Lanzhou 730070, China*

*^b^Gansu International Scientific and Technological Cooperation Base of Water-Retention Chemical Functional Materials, Lanzhou, 730070, China*

Synthesis of cubic CdS (C-CdS)

The cubic CdS were synthesized by a solvothermal method. Typically, Cd(CH_3_COO)_2_•2H_2_O (2.0 mmol) and thioacetamide (4 mmol) were first dissolved in deionized water (50mL) in a beaker placed on a magnetic stirrer for 30min at room temperature. Then, the mixture was transferred in a 100 mL Teflon-lined stainless-steel autoclave and heated at 180 ^◦^C for 24 h before cooling to room temperature. Then the yellow products were collected and washed by ethyl alcohol and deionized water three times. Finally, the products were dried in drying oven at 60°C for 4 h.


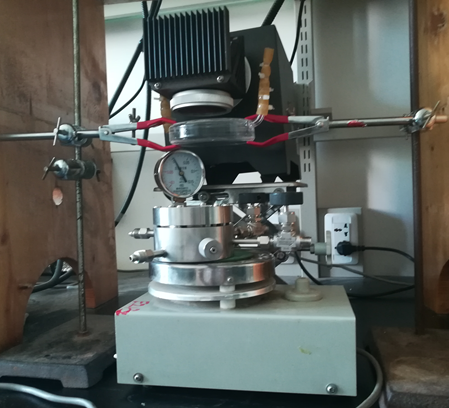


Figure S1The apparatus of photocatalytic reaction

Figure S2 (a) UV−vis diffuse reflectance spectra and (b) Tauc plots of C-CdS


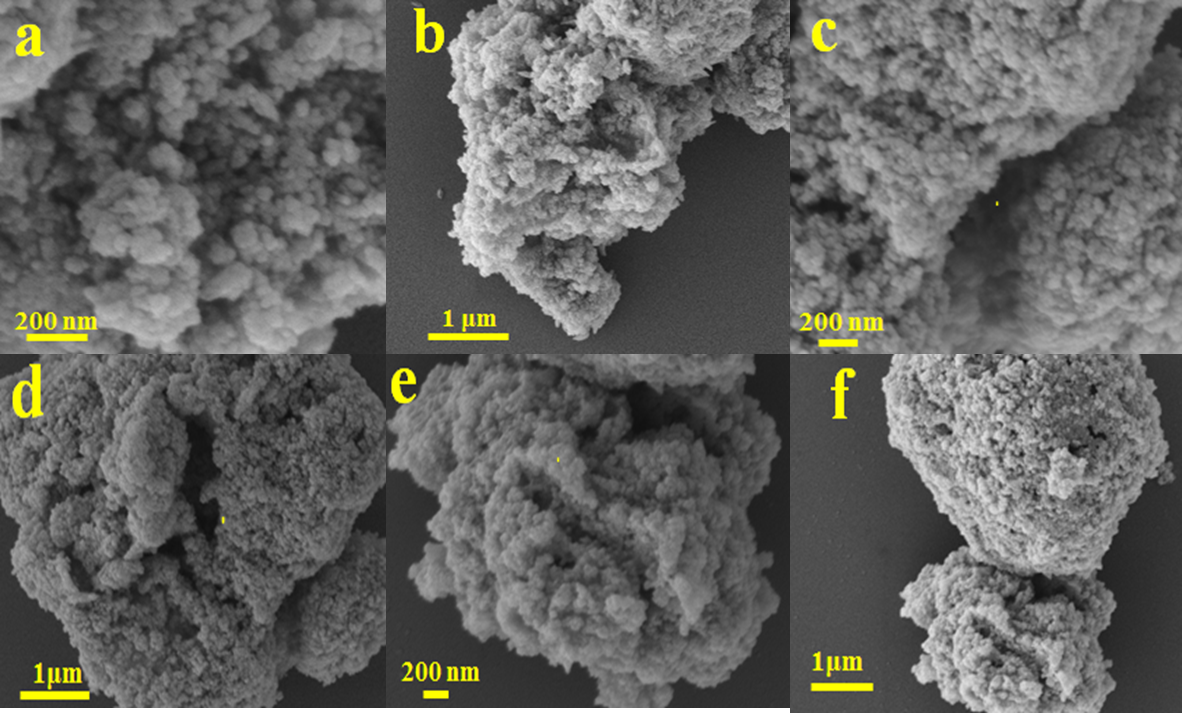


Figure S3 SEM images of 0.5wt%Ni(II)/CdS (a, b) ,1.0wt% Ni(II)/CdS (c,d) and 5.0wt%Ni(II)/CdS (e, f).

Figure S4 Mott-Schottky curves of 0.5wt%Ni(II)/CdS, 1.0wt%Ni(II)/CdS and 5.0wt%Ni(II)/CdS.


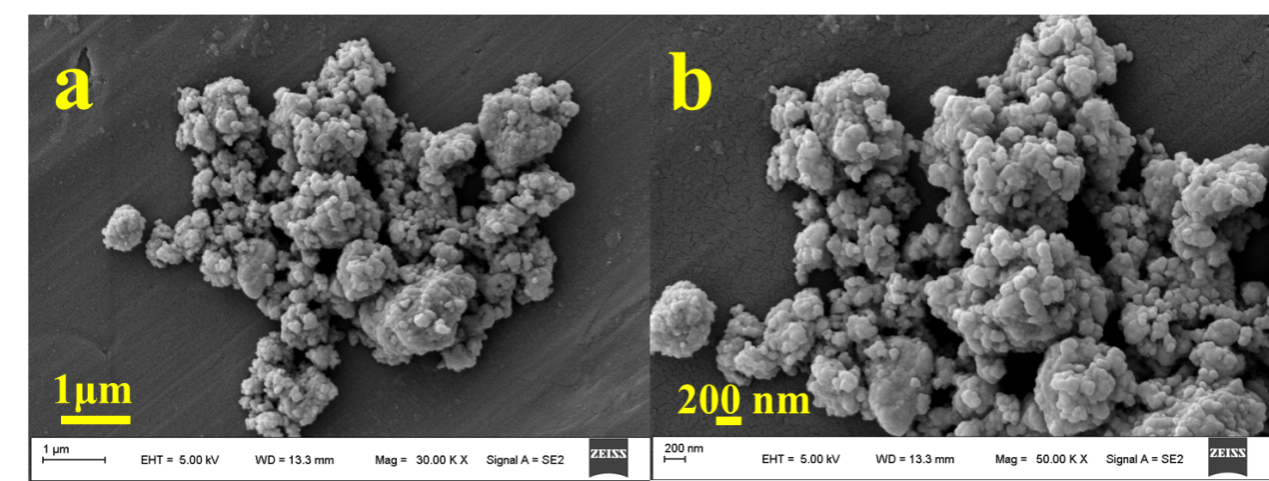


Figure S5 SEM images of used the 3.0wt%Ni(II)/CdS after photocatalytic reaction.

Figure S6 (a) XPS survey spectra and the high-resolution XPS spectra of (b) Cd 3d,

(c) S 2p, (d) Ni 2p for used the 3.0wt%Ni(II)/CdS.
